# Impaired balance between neutrophil extracellular trap formation and degradation by DNases in COVID-19 disease

**DOI:** 10.1186/s12967-024-05044-7

**Published:** 2024-03-07

**Authors:** Geoffrey Garcia, Sylvie Labrouche-Colomer, Alexandre Duvignaud, Etienne Clequin, Charles Dussiau, David-Alexandre Trégouët, Denis Malvy, Renaud Prevel, Atika Zouine, Isabelle Pellegrin, Julien Goret, Maria Mamani-Matsuda, Antoine Dewitte, Chloe James

**Affiliations:** 1 Biology of Cardiovascular Disease, INSERM, UMR 1034, Bordeaux University, CHU Haut-Lévêque, 1 Avenue Magellan, 33600 Pessac, France; 2 Laboratory of Hematology, Bordeaux University Hospital, 33600 Pessac, France; 3 Department of Infectious Diseases and Tropical Medicine, Hôpital Pellegrin, CHU Bordeaux, 33076 Bordeaux, France; 4 University Bordeaux, INSERM, Bordeaux Population Health Research Center, UMR 1219, 33000 Bordeaux, France; 5 CNRS, ImmunoConcEpT, UMR 5164, Inserm ERL1303, Bordeaux University, 33000 Bordeaux, France; 6 Department of Anaesthesia and Intensive Care, Bordeaux University Hospital, 33600 Pessac, France; 7 Medical Intensive Care Unit, Bordeaux University Hospital, 33000 Bordeaux, France; 8 Centre de Recherche Cardio-Thoracique de Bordeaux, INSERM, UMR 1045, Bordeaux University, 33000 Bordeaux, France; 9 CNRS, INSERM, TBM-Core, US5, UAR 3427, Flow Cytometry Facility, Bordeaux University, 33000 Bordeaux, France; 10 Centre de Ressources Biologiques, Bordeaux University Hospital, 33000 Bordeaux, France; 11 Department of Immunology and Immunogenetics, Bordeaux University Hospital, Bordeaux, France

## Abstract

**Background:**

Thrombo-inflammation and neutrophil extracellular traps (NETs) are exacerbated in severe cases of COVID-19, potentially contributing to disease exacerbation. However, the mechanisms underpinning this dysregulation remain elusive. We hypothesised that lower DNase activity may be associated with higher NETosis and clinical worsening in patients with COVID-19.

**Methods:**

Biological samples were obtained from hospitalized patients (15 severe, 37 critical at sampling) and 93 non-severe ambulatory cases. Our aims were to compare NET biomarkers, functional DNase levels, and explore mechanisms driving any imbalance concerning disease severity.

**Results:**

Functional DNase levels were diminished in the most severe patients, paralleling an imbalance between NET markers and DNase activity. DNase1 antigen levels were higher in ambulatory cases but lower in severe patients. DNase1L3 antigen levels remained consistent across subgroups, not rising alongside NET markers. *DNASE1* polymorphisms correlated with reduced DNase1 antigen levels. Moreover, a quantitative deficiency in plasmacytoid dendritic cells (pDCs), which primarily express *DNase1L3*, was observed in critical patients. Analysis of public single-cell RNAseq data revealed reduced *DNase1L3* expression in pDCs from severe COVID-19 patient.

**Conclusion:**

Severe and critical COVID-19 cases exhibited an imbalance between NET and DNase functional activity and quantity. Early identification of NETosis imbalance could guide targeted therapies against thrombo-inflammation in COVID-19-related sepsis, such as DNase administration, to avert clinical deterioration.

*Trial registration*: COVERAGE trial (NCT04356495) and COLCOV19-BX study (NCT04332016).

**Graphical Abstract:**

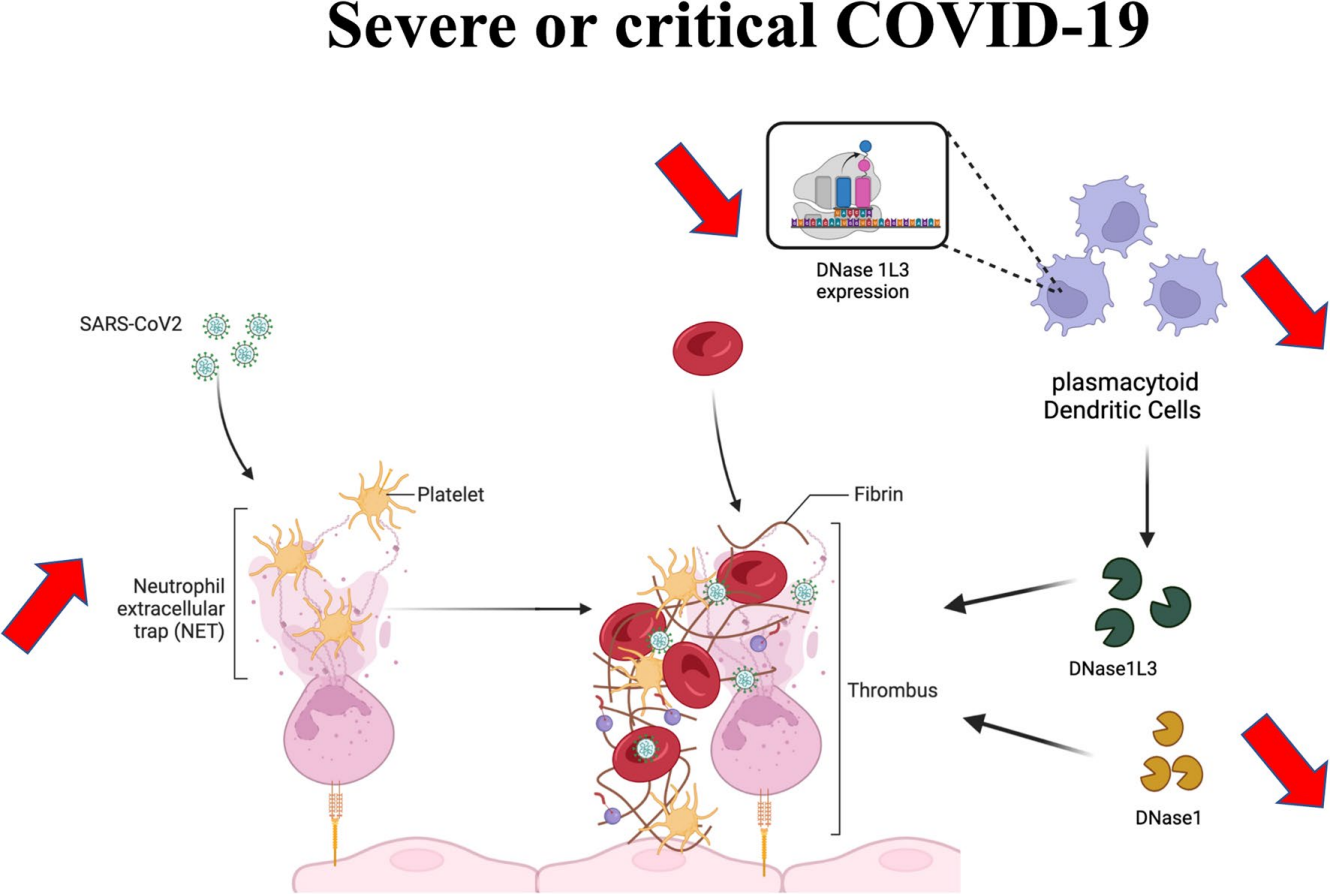

**Supplementary Information:**

The online version contains supplementary material available at 10.1186/s12967-024-05044-7.

## Introduction

The global health crisis instigated by the COVID-19 pandemic persists, resulting in a substantial toll with over 3.5 million recorded fatalities. The approximate case fatality rate for COVID-19 stands at about 1%, leading to hospitalization in 3–20% of cases [1], with a notable subset (approximately 10–30%) necessitating intensive care [2]. Most COVID-19 patients exhibit mild to moderate respiratory symptoms, including cough, fever, headache, myalgia, and occasionally diarrhea. Severe illness typically emerges approximately a week after the initial symptoms, characterized by dyspnea and progressive respiratory failure [3]. These patients often meet the criteria for Acute Respiratory Distress Syndrome (ARDS) [4]. Age, obesity, and male gender are widely recognized as significant risk factors for the development of severe COVID-19 [5]. Common comorbidities include hypertension, heart failure, cardiac arrhythmia, diabetes, kidney failure, and chronic pulmonary disease [6]. Furthermore, genetic predispositions for severe COVID-19 involve genes related to interferon signaling [7], and some older individuals harbor IFNα-neutralizing antibodies [8]. Nonetheless, the absence of specific COVID-19 therapies underscores our limited comprehension of the disease's pathogenesis, and there is still much to learn about predicting which patients will deteriorate and require intervention to prevent worsening.

A substantial body of evidence points to the involvement of thrombo-inflammation in the pathophysiology of COVID-19-related ARDS [9, 10]. Immunothrombosis represents a physiological innate immune response aimed at the formation of intravascular thrombi, intended to contain and eliminate pathogens, including bacteria, fungi, and viruses [11]. This intricate process implicates neutrophils, monocytes, platelets, and the activation of hemostatic pathways. Pathogen-triggered neutrophil activation triggers the release of neutrophil extracellular traps (NETs), composed of DNA filaments adorned with cytotoxic histones and enzymes such as myeloperoxidase (MPO) [12]. In vivo, NETs undergo degradation through two DNases, specifically DNase1 and DNase1-like 3 (DNase1L3) [13]. When unregulated, immunothrombosis poses a threat to the host. NETs exhibit procoagulant properties and are cytotoxic on pulmonary vascular endothelial cells [10]. Moreover, elevated NETosis has been implicated in various pathological processes, encompassing arterial and venous thrombosis [14].

Shortly after the emergence of the COVID-19 pandemic, speculations emerged regarding the potential role of uncontrolled immunothrombosis in driving disease progression towards severe forms in some patients. Microthrombi-induced vascular occlusion of lung capillaries and ensuing lung injury, along with macrothrombi contributing to the heightened incidence of thromboembolic events, were postulated as potential mechanisms [15]. The exact underlying reasons for the inefficient regulation of immunothrombosis remain elusive. Some investigators have reported the presence of anti-NET antibodies, which serve to stabilize NETs and hinder their clearance [16]. The absence of DNase1 and DNase1L3 has been demonstrated to result in severe vascular occlusions in a sepsis model [13], underscoring the critical importance of controlled NETosis.

In line with these considerations, our hypothesis was that a decline in DNase activity may be associated with augmented NETosis and clinical deterioration in COVID-19 patients. Our study's objectives were to examine the equilibrium between NETs and the functional capacity of DNase in patients spanning various degrees of COVID-19 severity and to investigate the underlying factors contributing to potential deficiencies in DNase functional activity.

## Results

### Patient characteristics

Table 1 summarizes the characteristics and outcomes of the study participants. A total of 145 COVID-19 patients were enrolled, with 93 in the non-severe outpatient group and 52 in the inpatient group, which included 15 individuals with severe disease and 37 with critical disease upon admission. Among the critical COVID-19 patients, 32 were hospitalized in an intensive care unit, while 5 were admitted to conventional wards. The 15 patients with severe COVID-19 were hospitalized in conventional wards. Non-severe outpatients received no specific treatment for COVID-19, except a regimen of vitamins and trace elements at physiological doses. In contrast, all critical patients received dexamethasone, whereas only eight (53%) of severe patients received this treatment (P < 0.0001). Tocilizumab was administered to eight (22%) critical patients, while only one (7%) severe patient received it (P < 0.0001). Only one patient in the critical group and one in the severe group received antiviral therapy with remdesivir. Notably, neither non-severe outpatients nor severe inpatients developed thrombotic complications, in contrast to three (8%) critical patients (P = 0.1). Critical patients also exhibited a higher incidence of sepsis compared to severe patients (41% vs. 13%; P < 0.0001). Ten non-severe patients were eventually admitted to the hospital during follow-up, and two of them succumbed to the disease.

**Table 1:** Patients’ characteristics and outcomes

	Non-severe COVID-19 (n = 93)	Severe COVID-19 (n = 15)	Critical COVID-19 (n = 37)	P value
Demographics				
Age (years)	63 [60–69]	58 [43–69]	64 [59–77]	0.1
Gender (male)	46 (49)	10 (67)	30 (81)	0.003
Body mass index (BMI)	27 [23–29]	25 [13, 22–27]	29 [25–31]	0.04
Comorbidities				
Hypertension	41 (44)	2 (13)	23 (62)	0.005
Diabetes	16 (17)	0 (0)	10 (27)	0.02
Obesity	22 (24)	2 (13)	14 (41)	0.1
Cardiovascular disease	14 (15)	1 (7)	5 (14)	0.8
Chronic respiratory disease	5 (5)	5 (33)	9 (24)	0.0008
Stage 3 chronic kidney disease	0 (0)	1 (7)	5 (14)	0.002
I mmunosuppressive treatments	0 (0)	0 (0)	1 (3)	0.04
Solid tumors or hematological malignancies	4 (4)	0 (0)	1 (3)	0.7
Inclusion characteristics				
Symptoms to inclusion time	0 [0–1]	2 [1, 2]	7 [3–9]	< 0.0001
Temperature at inclusion (°)	36.8 [36.3–37.7]	37.3 [36.7–38.1]	37.7 [37–38.4]	0.0002
SOFA score	NA	1 [1, 2]	3 [2–4]	0.001
IGS II score	NA	15 [9–22]	30 [27–32]	< 0.0001
CRP (mg/L)	7 [4–14]	52 [33–140]	134 [75–215]	< 0.0001
Platelets(G/L)	207 [171–237]	302 [191–340]	250 [202–338]	0.0002
Neutrophils (/mm3)	2.6 [2.1–3.5]	4.6 [2.1–3.5]	6.5 [4.5–10.4]	< 0.0001
Lymphocytes (/mm3)	1.4 [1–1.8]	0.8 [0.7–2.1]	0.6 [0.4–0.8]	< 0.0001
Neutrophils/Lymphocytes ratio	2 [1.4–2.8]	2.4 [1.7–7.1]	11.0 [6.9–22.4]	< 0.0001
Platelets/Lymphocytes ratio	158 [110–205]	219 [149–442]	402 [252–570]	< 0.0001
PaO2/FiO2 ratio	NA	270 [236–306]	157 [113–198]	0.004
ROX index	NA	16 [12–20]	7 [5–9]	< 0.0001
Percentage of lung injury^a^	NA			< 0.0001
10–25%		8 (62)	5 (16)	
25–50%		4 (31)	13 (42)	
50–75%		1 (8)	10 (32)	
75–100%		0 (0)	3 (10)	
Specific CoVID-19 treatment				
Corticosteroids	0 [0–0]	8 (53)	37 (100)	< 0.0001
Tocilizumab	0 [0–0]	1 (7)	8 (22)	< 0.0001
Respiratory support				
Standard oxygen therapy	0 (0)	15 (100)	0 (0)	< 0.0001
High flow nasal oxygen	0 (0)	0 (0)	30 (81)	< 0.0001
Invasive ventilation	0 (0)	0 (0)	7 (19)	< 0.0001
Prone ventilation	0 (0)	0 (0)	7 (19)	< 0.0001
Anticoagulation therapy				
Prophylactic anticoagulation	0 [0–0]	9 (60)	13 (31)	< 0.0001
Therapeutic anticoagulation	0 [0–0]	6 (40)	24 (69)	< 0.0001
Outcomes				
Thrombotic event	0 [0–0]	0 (0)	3 (8)	0.01
Sepsis occurrence	0 [0–0]	2 (13)	15 (41)	< 0.0001
Length of stay in the ICU	NA	0 [0–0]	8 [6–16]	< 0.0001
Length of stay in the hospital	NA	9 [5–10]	19 [11–24]	< 0.0001
Mortality	2 (2)	0 (0)	5 (14)	0.02

Continuous quantitative variables are expressed as median and interquartile range. Categorical variables are expressed as frequencies and proportions

*NA* Not available

^a^Percentage of lung lesion on first computed tomography (CT)

P value for comparison between groups (Kruskal–Wallis for quantitative variable or Pearson chi-squared test for qualitative variables)

### NET biomarkers and disease severity

**Fig. 1:**
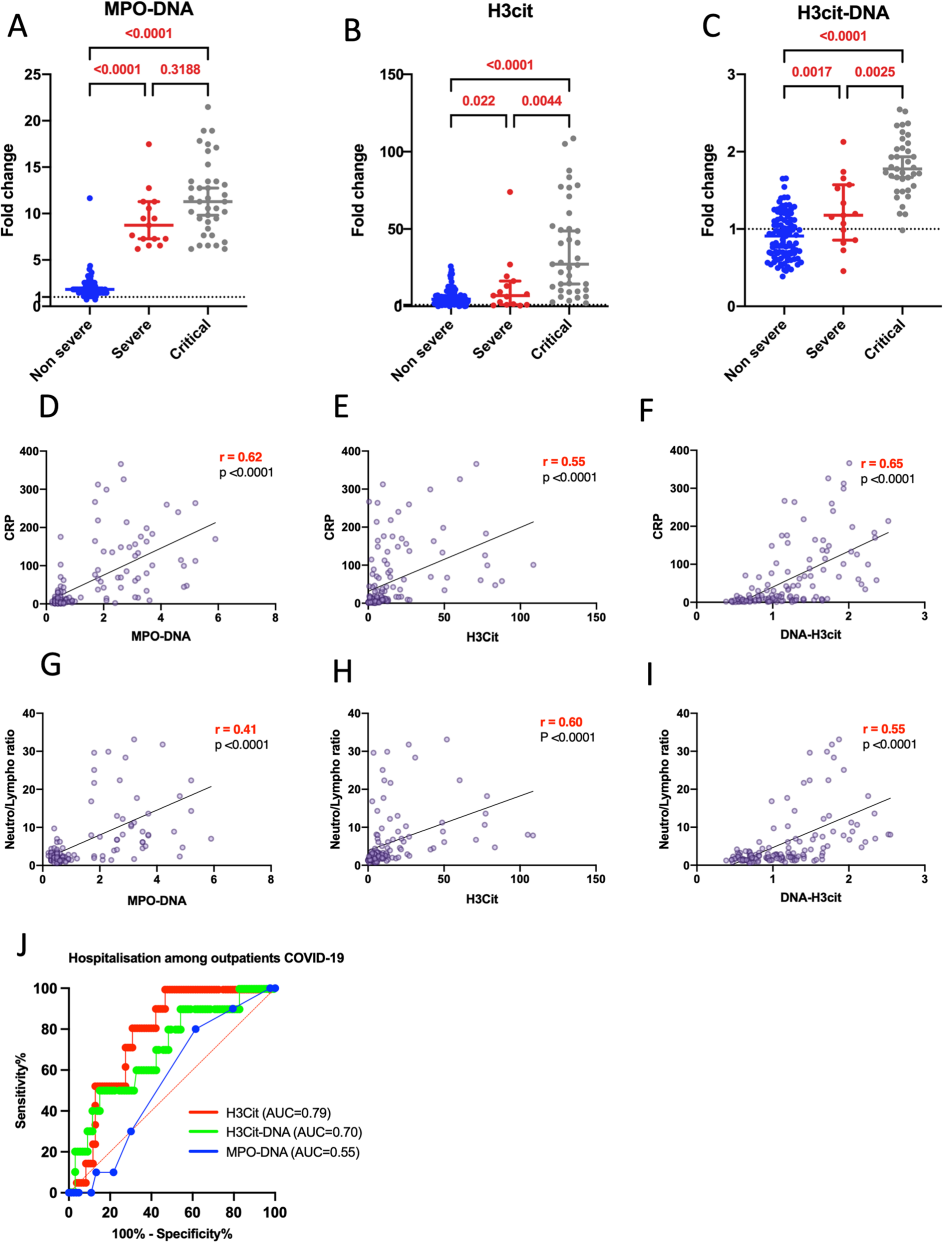
Plasma NETs markers correlate with COVID 19 clinical severity, biological markers of disease severity and predict hospitalization in non-severe patients. **A**–**C** We compared three NET biomarkers, MPO-DNA (**A**), H3cit (**B**), and H3cit-DNA complexes (**C**) between non-severe (oupatients) (n = 93), severe (without needs of oxygen therapy or with standard oxygen therapy) (n = 15) and critical COVID-19 patients (with high flow nasal oxygen, invasive intubation or prone ventilation) (n = 37). Results are expressed as fold change compared to healthy donors (n = 21). Statistical evaluation was performed using a Compound Poisson-Gamma model adjusted for age, sex and BMI. **D**–**I** Spearman correlation between three NET biomarkers, MPO-DNA (**D**, **G**), H3cit (**E**, **H**), and H3cit-DNA complexes (**F**, **I**) and two biological disease severity markers, *i.e.* CRP (**D**–**F**) and neutrophils/lymphocytes ratios (**G**–**I**) across all COVID-19 patients (n = 145). Statistical analysis are adjusted for age, gender and BMI, after log transformation of CRP variable. **J**–**K** Receiver-operating characteristic curve for prediction of hospitalization in COVID-19 outpatients using MPO–DNA, H3cit and H3cit-DNA complexes levels (**J**)

We measured NET biomarkers in plasma collected at the time of inclusion, i.e. before administration of any treatment for non-severe outpatients, and at the time of hospital admission for severe and critical inpatients. All results described thereafter were obtained after adjusting for age, gender and Body-Mass-Index (BMI), the most important known risk factors for COVID-19 aggravation. The elevation of NET biomarkers, as determined by MPO-DNA, H3cit, and H3cit-DNA complexes, was more pronounced in critical patients compared to outpatients (Fig. 1A–C). This increase was directly proportional to disease severity. Additional file 1: Figure S1 presents cfDNA values, which displayed a similar trend. In conjunction with this finding, we observed that MPO-DNA, H3cit, and H3cit-DNA complexes correlated with CRP (Fig. 1D–F) and the neutrophil/lymphocyte ratio (Fig. 1G–I), both being key biological markers of disease severity in all COVID-19 patients [17].

Additional file 1: Figure S2 illustrates the correlation between NET biomarkers and two clinical markers of disease severity, namely the PaO2/FiO2 ratio (Additional file 1: Figure S2A-C) and the ROX index (Additional file 1: Figure S2D-F). Both of them were only applicable to hospitalized patients. The PaO2/FiO2 ratio is a widely used clinical indicator of hypoxemia known to correlate with mortality [4] whereas the ROX index, which is the ratio of oxygen saturation measured by pulse oximetry/FiO2 to respiratory rate, was employed to identify patients at low or high risk for intubation [18]. No correlations between NETs markers and the PaO2/FiO2 ratio were observed but all NETs markers correlated negatively with the ROX index (Additional file 1: Figure S2). Notably, the levels of two NET markers, H3cit and H3cit-DNA complexes, were predictive of hospitalization among outpatients with non-severe COVID-19 (Fig. 1J). In summary, these results suggest a clear association between the levels of NET biomarkers and disease severity, leading to adverse outcomes in COVID-19 patients.

### Insufficient functional dnase in severe and critical patients

**Fig. 2:**
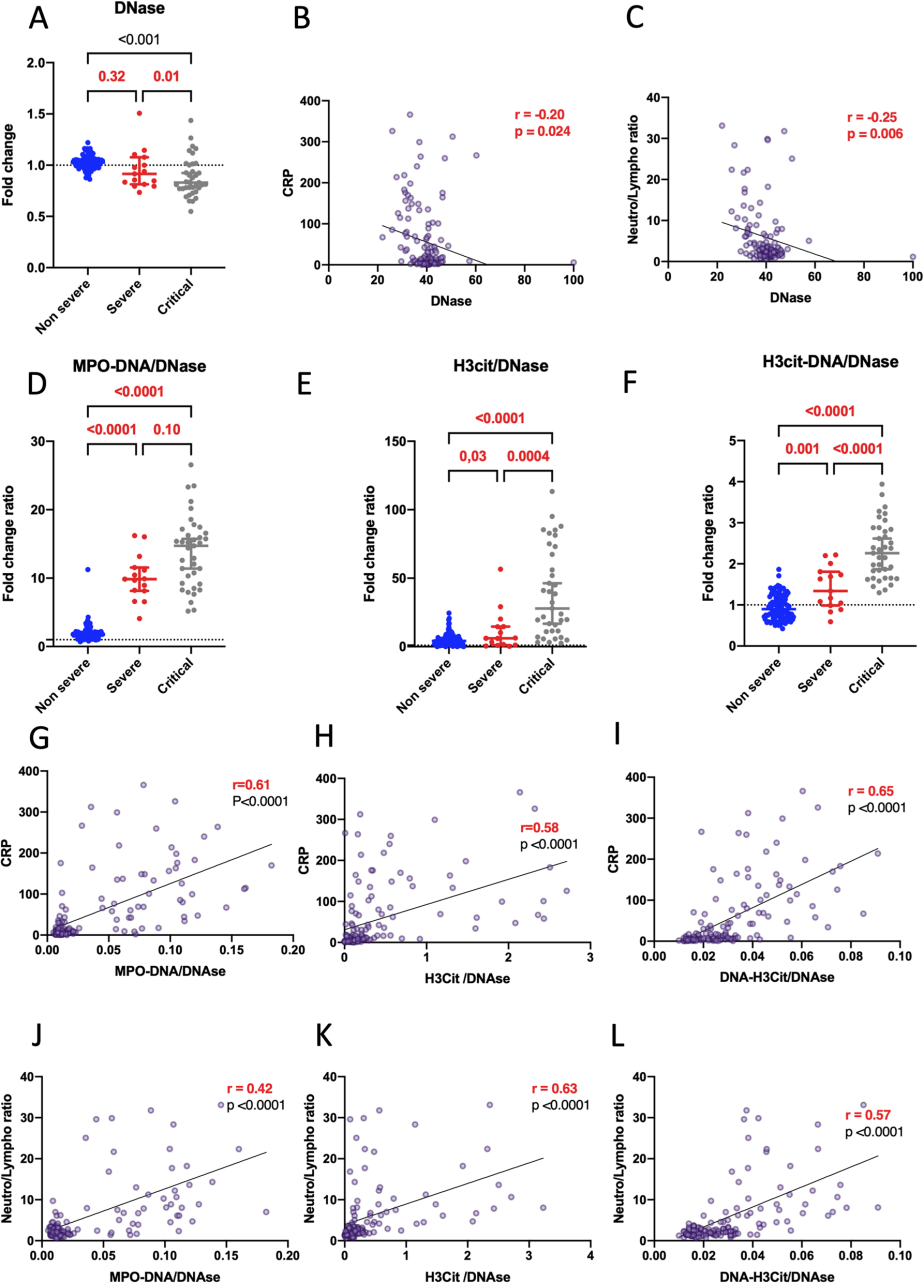
The level of functional DNase is lower in hospitalized patients compared to outpatients, resulting in an imbalanced NET markers/DNase ratio. **A** We examined the level of functional DNase (DNase) between non-severe (n = 93), severe (n = 15) and critical COVID-19 patients (n = 37). Results are expressed as fold change compared to healthy donors (n = 21). **B**, **C** Spearman correlation explores the relationship between the level of functional DNase and two disease severity markers, CRP (**B**), and neutrophils/lymphocytes ratio (**C**) across all patients (n = 145). **D**–**F** We compared the ratio between NET biomarkers [MPO-DNA (**D**), H3cit (**E**), and H3cit-DNA (**F**)] and DNase between non-severe (n = 93), severe (n = 15), and critical COVID-19 patients (n = 37) Results are expressed as a fold change ratio. **G**–**L** Spearman correlation investigates the correlation between the ratio of NET markers [MPO-DNA (**G**, **J**), H3cit (**H**, **K**), and H3cit-DNA (**I**, **L**)] over DNase and two severity markers, CRP (**G**–**I**), and neutrophils/lymphocytes ratio (**J**–**L**) in all COVID-19 patients (n = 145). Comparisons between groups were performed using a Compound Poisson-Gamma model adjusted for age, sex and BMI. Correlation were realized using Spearman method, adjusted for age, gender and BMI, after log transformation of CRP variable

Considering that NETs are physiologically degraded by DNases, we postulated that the balance between NET production and degradation might be disrupted in severe patients. Consequently, we aimed to quantify the functional DNase in our cohort and devised an assay to measure total DNase functional activity by assessing residual double-stranded DNA (dsDNA) after incubation with plasma samples. Our observations revealed functional DNase remained consistent in non-severe COVID-19 patients compared to healthy donors. In contrast, the functional DNase level was lower in severe and critical COVID-19 patients compared to non-severe COVID-19 patients (Fig. 2A). Furthermore, DNase exhibited an inverse correlation with CRP and the neutrophil/lymphocyte ratio (Fig. 2B, C). To delve deeper into the balance between NET production and degradation, we examined the ratio between NET biomarkers and the functional DNase. These ratios, encompassing cfDNA, MPO-DNA, H3cit, H3cit-DNA to DNase, were markedly higher in the most severe patients (Fig. 2D–F and Additional file 1: Figure S1) and demonstrated significant correlations with markers of disease severity, ie CRP (Fig. 2G–I) and neutrophil/lymphocyte ratio (Fig. 2J–L). These ratios also tended to negatively correlated with the ROX index in hospitalized patients, as illustrated in Additional file 1: Figure S3. Altogether, the results underscore a notable imbalance between NET production and degradation in severe patients, particularly in the most critical cases.

### Insufficient increase in DNase1 and DNase1L3 in severe and critical patients

**Fig. 3:**
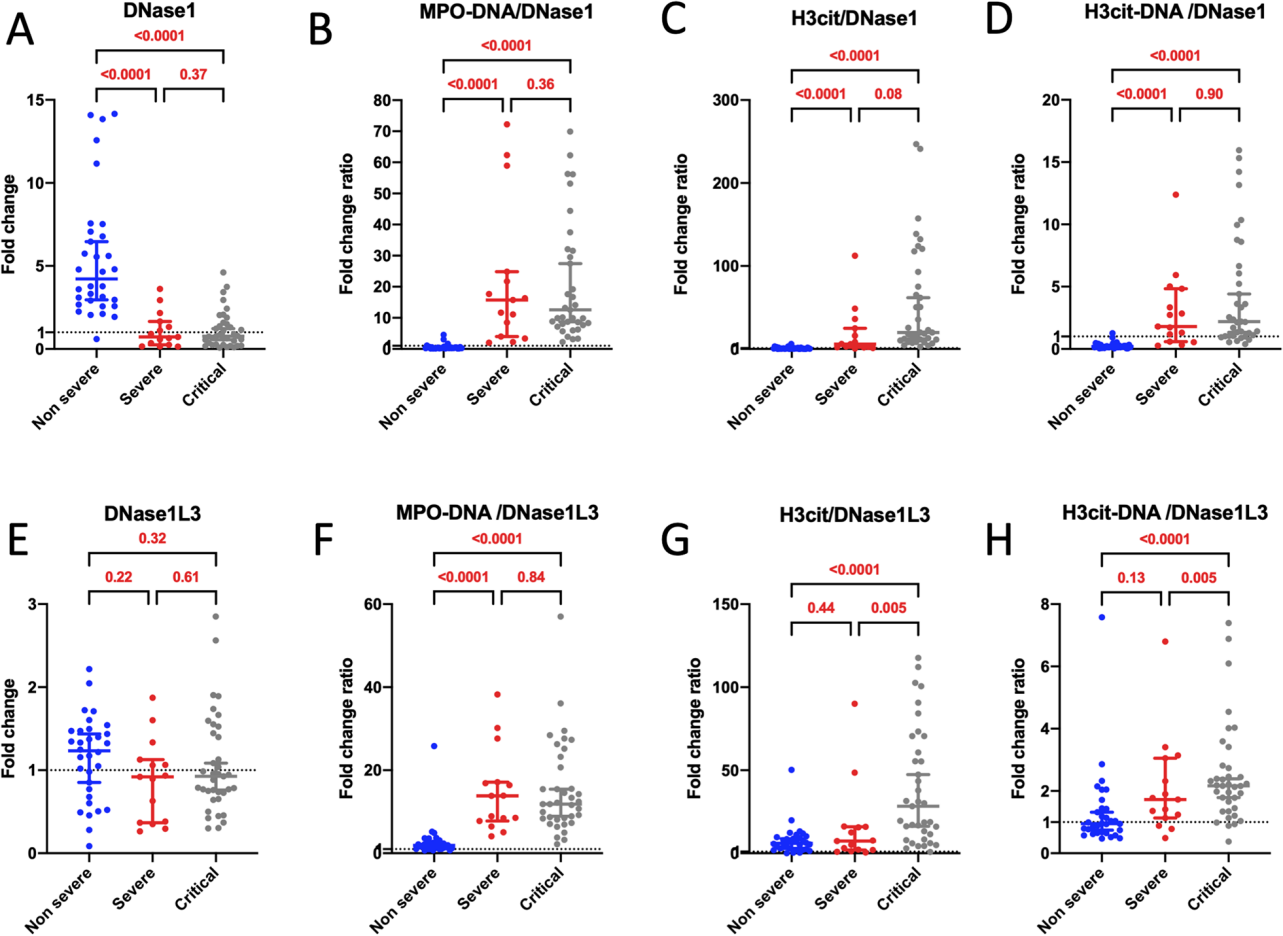
The equilibrium between NET biomarkers and DNase1 or DNase1L3 antigens is disrupted in severe patients. **A**–**D** Comparison of the quantity of DNase1 protein (**A**) and the ratios between NET biomarkers [MPO-DNA (**B**), H3cit (**C**), H3cit-DNA (**D**)] and DNase1 antigen among non-severe (n = 32), severe (n = 15), and critical COVID-19 patients (n = 37). Results are expressed as fold change in comparison to healthy donors (n = 7) and fold change ratio. **E**–**H** Comparison of the quantity of DNase1L3 protein (**E**) and the ratios between NET biomarkers [MPO-DNA (**F**), H3cit (**G**), and H3cit-DNA (**H**)] and DNase1L3 antigen among non-severe (n = 32), severe (n = 15), and critical (n = 37) COVID-19 patients. Results are expressed as fold change in comparison to healthy donors (n = 7) and fold change ratio. Statistical analyses were conducted using a Compound Poisson-Gamma model adjusted for age, sex and BMI

Our next investigation aimed to elucidate why functional DNase levels were lower in severe and critical COVID-19 patients compared to non-severe patients. Our technique for measuring functional DNase levels assessed both DNase1 and DNase1L3's ability to degrade dsDNA. We thus quantified the amount of DNase1 and DNase1L3 proteins amount via ELISA in patients' plasma samples. Intriguingly, we observed an elevated quantity of DNase1 protein in outpatients compared to healthy donors, while a lower amount was found in severe and critical patients compared to non-severe outpatients (Fig. 3A). The ratios between all three NET markers and DNase1 antigen were also notably higher in the most severe patients (Fig. 3B–D), further supporting the idea of an impaired balance between DNase1 protein and NET markers in the most severe COVID-19 patients. Notably, no significant differences were observed in DNase1L3 ELISA in outpatients compared to healthy donors and severe patients compared to non-severe patients (Fig. 3E). However, akin to DNase1 antigen, the balance between NET markers and the amount of DNase1L3 antigen was higher in severe and critical patients compared to non-severe patients (Fig. 3F–H). Collectively, it appeared that circulating DNase1 and DNase1L3 were not sufficiently upregulated to eliminate the NETs formed in severe and critical COVID-19 patients.

### DNASE1 polymorphisms and DNase1 protein level

A pursuit to uncover why circulating DNase levels did not increase in proportion to NETosis during severe or critical COVID-19 led to the hypothesis that genetic variations in *DNASE1* and *DNASE1L3* genes might be responsible for the deficiency in DNase amount or function. This led to an exploration of genetic polymorphisms at *DNASE1L3* and *DNASE1* in hospitalized patients (Tables 2 and 3, respectively). All identified variations, whether rare or common, are listed in Additional file 1: Tables S4 and S5. Notably, no variations were detected in *DNASE1* coding regions. Common polymorphisms were assessed for associations with the levels of encoded proteins and the results are outlined in Tables 2 and 3 for DNase1L3 and DNase1 antigens, respectively. While no associations at P < 0.05 were observed between *DNASE1L3* polymorphisms and DNase1L3 antigen (Table 2), a set of nine polymorphisms (rs45564535, rs45606645, rs45626736, rs865833716, rs867920095, rs77563984, rs79356805, rs17136471, and rs8176922), all displaying strong linkage disequilibrium (r^2^ ~ 1), exhibited evidence (P ~ 0.01) of association with DNase1 antigen (Table 3). Notably, these polymorphisms were associated with a ~ 75% reduction in DNase1 antigen, and all three carriers of the minor allele for these polymorphisms were critical patients.

**Table 2:** Association of common DNASE1L3 polymorphisms with DNAse1L3 level in hospitalized patients

rsID	Genomic position (GRCh38)	Alleles^a^	Amino acid position	Localization	MAF^b^	Effect on DNase1L3 protein level^c^			P^d^
						Homozygous (ref allele)	Heterozygous	Homozygous (alternative allele)	
rs2292677	58210912	c.-6G > A		5’UTR	0.375	0.91 (0.73–1.35) n = 20	0.93 (0.64–1.13) n = 25	0.77 (0.32 –1.88) n = 7	0.75
rs34252389	58210856	c.51C > T		Exon 1	0.038	0.91 (0.60–1.18) n = 48	1.24 (0.85–1.56) n = 4	NA	0.49
rs3732631	58208324	c.142-18G > T		Intron 1	0.375	0.83 (0.65–1.35) n = 20	0.94 (0.64–1.13) n = 25	0.88 (0.63–1.88) n = 7	0.73
rs74350392	58205547	c.244G > C	p.Gly82Arg	Exon 3	0.029	0.93 (0.65–1.40) n = 49	0.45 (0.40–0.61) n = 3	NA	0.72
rs2070117	58205539	c.252G > A	p.Thr84 =	Exon 3	0.221	0.95 (0.75–1.59 n = 33	0.88 (0.39–1.06) n = 15	0.69 (0.57–0.81) n = 4	0.12
rs17058970	58205515	c.276G > C	p.Arg92 =	Exon 3	0.058	0.92 (0.57–1.42) n = 47	0.87 (0.77–0.99) n = 4	0.75 n = 1	0.69
rs3772985	58200992	c.546 + 5G > T		Intron 5	0.115	0.92 (0.71–1.41) n = 40	0.85 (0.45–1.18) n = 12	NA	0.37
rs35677470	58197909	c.616C > T	p.Arg206Cys	Exon 6	0.067	0.93 (0.65–1.28) n = 46	0.92 (0.65–1.89) n = 5	3.3 n = 1	0.48
rs113844064	58197694	c.704 + 127G > T		Intron 6	0.040	0.92 (0.64–1.23) n = 47	0.93 (0.61–1.24) n = 2	0.77 n = 1	0.50
rs3732630	58193301	c.801 + 42A > G	p.Lys281 =	Intron 7	0.360	1.01 (0.74–1.42) n = 30	0.78 (0.48–0.96) n = 16	0.76 (0.38–1.53) n = 5	0.21
rs144058112	58193270	c.801 + 73 T > C		Intron 7	0.080	0.92 (0.63–1.40) n = 45	1.03 (0.76–1.29) n = 2	0.88 (0.83–0.91 n = 3	0.49
rs3732629	58193139	c.801 + 204G > A		Intron 7	0.281	0.93 (0.73–1.39) n = 27	0.77 (0.46–0.96) n = 15	0.91 (0.48–1.41) n = 6	0.13
rs34882513	chr3:58192419	c.*268delC		3' UTR	0.067	0.89 (0.50–1.13) n = 45	0.93 (0.78–1.44) n = 7	NA	0.76

^a^Nomenclature according to NM_004944.4 reference

^b^Minor Allele Frequency observed in our cohort (n = 52)

^c^Data are represented as median (interquartile range 1—interquartile range 3) of DNase1L3 fold change

^d^Association test ‘s p values obtained using linear regression analysis on log(DNase1L3 fold change) activity under the assumption of dominant genetic effects, adjusted for age and sex

**Table 3:** Association of DNASE1 polymorphisms with DNase1 level in hospitalized patients

rsID	Genomic position (GRCh38)	Alleles^a^	Amino Acid position	Localization	MAF^b^	Effect on DNase1 protein level^c^			P^d^
						Homozygous (ref allele)	Heterozygous	Homozygous (alternative allele)	
rs45564535	3653249	c.-1797A > C	NA	Upstream transcript	0.029	0.76 (0.35–1.58) n = 49	0.19 (0.14–0.54) n = 3	NA	0.0155
rs45606645	3653474	c.-1572 T > C	NA	Upstream transcript	0.029	0.76 (0.35–1.58) n = 49	0.19 (0.14–0.54) n = 3	NA	0.0155
rs45626736	3653508	c.-1538G > A	NA	Upstream transcript	0.0288	0.76 (0.35–1.58) n = 49	0.19 (0.14–0.54) n = 3	NA	0.0155
rs865833716	3653840	c.-1206A > C	NA	Upstream transcript	0.0294	0.77 (0.36–1.60) n = 48	0.19 (0.14–0.54) n = 3	NA	0.0132
rs867920095	3653841	c.-1205A > G	NA	Upstream transcript	0.030	0.78 (0.43–1.62) n = 47	0.19 (0.14–0.54) n = 3	NA	9.55 10^–3^
rs77563984	3653879	c.-1167C > T	NA	Upstream transcript	0.03	0.79 (0.43–1.62) n = 47	0.19 (0.14–0.54) n = 3	NA	9.55 10^–3^
rs79356805	3653880	c.-1166C > G	NA	Upstream transcript	0.03	0.79 (0.43–1.62) n = 47	0.19 (0.14–0.54) n = 3	NA	9.55 10^–3^
rs17136471	3654596	c.-450G > A		Upstream transcript	0.0385	0.77 (0.33–1.60) n = 48	0.41 (0.17–0.70) n = 4	NA	0.0158
NA	3654725_ 3654729	c.-317_-313del		5’UTR	0.0385	0.76 (0.30–1.52) n = 48	0.74 (0.46–1.61) n = 4	NA	0.860
rs117176134	3655097	c.-2 + 54G > C		Intron 1	0.0288	0.75 (0.25–1.37) n = 49	2.07 (1.37–2.91) n = 3	NA	0.341
rs8176922	3655358	c.-1-15C > T		Intron 1	0.0288	0.76 (0.35–1.58) n = 49	0.19 (0.14–0.54) n = 3	NA	0.0155
rs146417970	3655625	c.147 + 105 T > C		Intron 2	0.0288	0.76 (0.27–1.58) n = 49	0.57 (0.41–0.68) n = 3	NA	0.485
rs181314680	3655818	c.148-31C > G		Intron 2	0.0288	0.78 (0.27–1.58) n = 49	0.62 (0.43–0.63) n = 3	NA	0.105
rs1799892	3657408	c.704 + 67G > C		Intron 7	0.4231	0.75 (0.26–1.69) n = 19	0.83 (0.58–1.53) n = 22	0.55 (0.21–1.18) n = 11	0.164
rs1053874	3657746	c.731G > A	p. (Arg244Gln)	Exon 8	0.43	0.74 (0.31–2.02) n = 20	0.90 (0.58–1.33) n = 17	0.58 (0.24–1.37) n = 13	0.175

^a^Nomenclature according to NM_005223.4 reference

^b^Minor Allele Frequency observed in our cohort (n = 52)

^c^Data are represented as median (interquartile range 1—interquartile range 3) of DNase1 fold change

^d^Association test ‘s pvalues obtained using compound Poisson Gamma model on DNase1 fold change level under the assumption of dominant genetic effects, adjusted for age and sex

### Reduced plasmacytoid dendritic cells and DNase1L3 RNA expression in severe patients

**Fig. 4:**
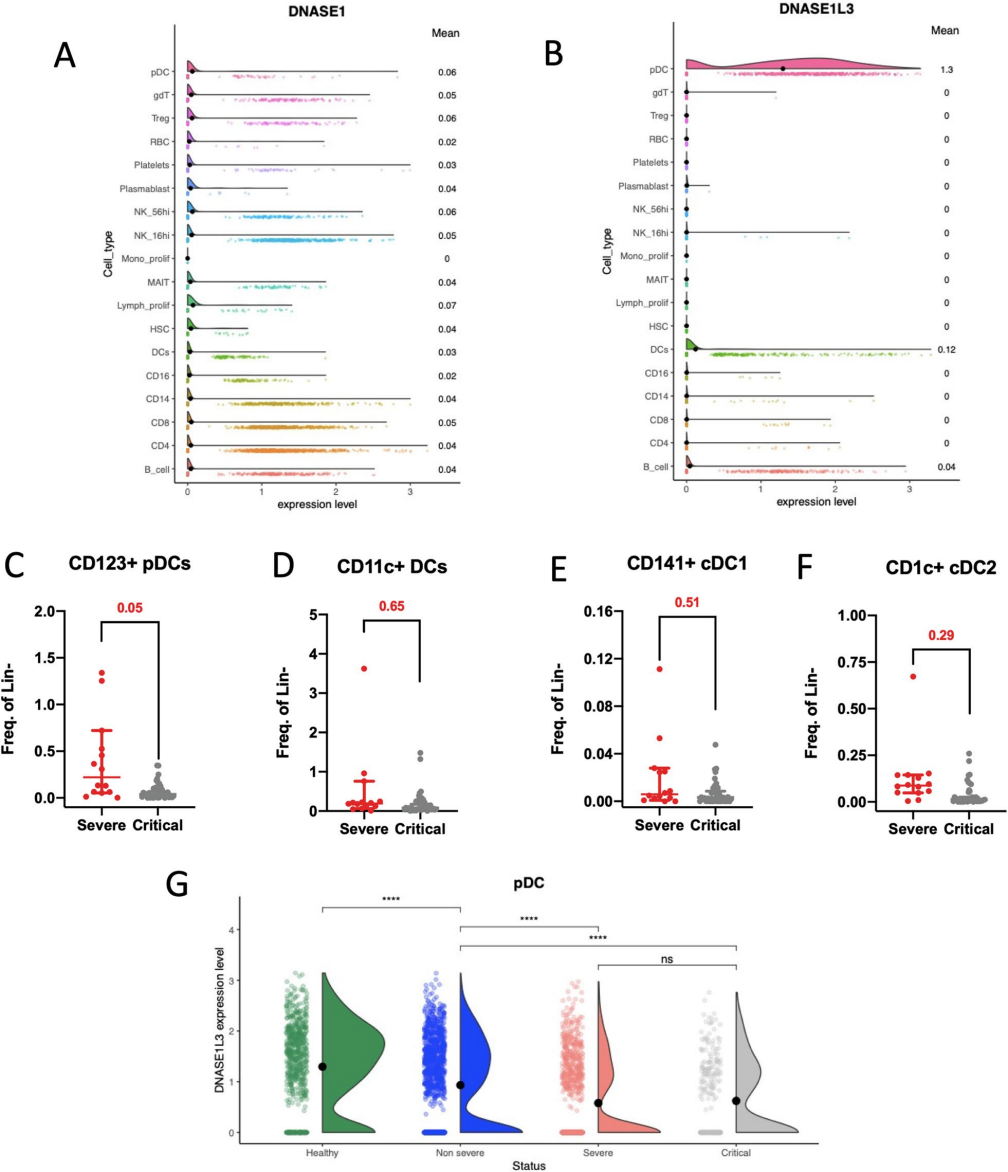
Defective dendritic cells and plasmacytoid dendritic cells in severe COVID-19 patients. **A**, **B** Violin plot displaying normalized gene expression levels of *DNASE1* (**A**) and *DNASE1L3* (**B**) in blood cells from healthy donors (n = 24) (data analysis from Stephenson E. and al (19)). Means are indicated on the right. **C**–**F** Compound Poisson-Gamma model adjusted for age, sex and BMI compared the count of CD123 + pDCs (**C**), CD11c + DCs (**D**), CD141 + cDC1 (**E**), and CD1c + cDC2 (**F**) between severe (n = 27) and critical (n = 37) patients. Results are expressed as frequency of alive lineage negative cells. The horizontal line defines median value. (**G**) Violin plot showing *DNASE1L3* normalized gene expression in pDC from healthy donor (n = 24), non-severe (n = 25), severe (n = 15) and critical (n = 16) COVID-19 patient (data analysis from Stephenson et al. [19]). Statistical analyses were conducted using the Kruskal–Wallis test with Dunn’s multiple comparisons

Our investigation extended to understanding why circulating DNase1 and DNase1L3 were not efficiently upregulated during severe and critical COVID-19. We theorized that cells responsible for producing circulating DNase1 and DNase1L3 were deficient. Our analysis of publicly available single-cell RNAseq data from healthy donors [19] unveiled *DNASE1* and *DNASE1L3* expression in various blood cell populations (Fig. 4A, B). Notably, *DNASE1* expression was generally low in all blood cells (Fig. 4A), while *DNASE1L3* was primarily expressed in plasmacytoid dendritic cells (pDCs) and to a lesser extent in dendritic cells (DCs) (Fig. 4B). We quantified pDCs (CD11c-CD123 +) and DCs (CD11c + CD123-/dim) in severe and critical inpatients using flow cytometry (Fig. 4C–F and Additional file 1: Figure S4). Among DCs, we differentiated conventional dendritic cells 1 (cDC1s: CD141 + , CD1c-) and conventional dendritic cells (cDC2s: CD141-/dim, CD1c +) (Additional file 1: Figure S4). A lower number of pDCs was observed in critical patients compared to severe patients (Fig. 4C–F). Our analysis also explored the possibility that pDCs and DCs might be dysfunctional in the most severe patients. We investigated *DNASE1L3* gene expression in pDCs and DCs using publicly available single-cell RNAseq data from 80 COVID-19 patients [19]. We categorized them into three groups based on disease severity with the same criteria for severity we used for the 145 patients we prospectively included. We observed a decline in *DNASE1L3* expression in pDCs as disease severity increased (Fig. 4G), although no differences were observed in DCs. Altogether, our findings suggest that severe COVID-19 is linked to defects in DCs and pDCs, which may explain the inadequate production of DNase1L3 needed to clear NET complexes.

## Discussion

This translational study investigates the equilibrium between NETosis and its regulation by DNases in a diverse population of COVID-19 patients, including outpatients without hospital admission criteria or the need for oxygen supplementation. The study is based on samples prospectively collected from patients participating in clinical studies. Our study demonstrates that elevated NETs markers and reduced functional DNases are risk factors for COVID-19 severity, independently of age, gender and BMI, the major determinants of severe COVID-19.

We found a strong correlation between NET markers and disease severity, in line with previous reports (as reviewed by Bonaventura A. et al. [10] and our own findings [20]). Currently, there is no standardized reference test for NET quantification, but efforts are underway to establish recommendations for NET measurement standardization through the Scientific and Standardization Subcommittee of the International Society of Thrombosis and Haemostasis. To enhance the robustness of our results, we utilized three contemporary NET plasma markers: MPO-DNA, H3Cit, and H3Cit-DNA complexes. Although cfDNA is considered a non-specific marker for NETs, as it also measures DNA from necrotic cells [21], it displayed a similar trend in our study. Notably, markers involving H3Cit quantification are regarded as the most specific. Our results were largely concordant across the three NET markers, reinforcing our confidence in the observations.

The reason behind the escalation of NETs with disease severity has recently come under scrutiny. NET degradation was found to be compromised in adult COVID-19 patients [22], with more pronounced impairment in symptomatic patients compared to asymptomatic cases at diagnosis. Moreover, NET degradation improved with disease recovery, indicating a direct correlation between intravascular NET degradation impairment and disease severity. Some COVID-19 patients were identified as having anti-NET antibodies that hinder the ability of healthy control serum to degrade NETs [16]. These antibodies might stabilize NETs and hinder their clearance. It is also plausible that high levels of NETs lead to a reduced availability of DNase.

Intravascular NETs are primarily degraded by endogenous DNases, specifically DNase1 and DNase1L3 [13]. Thus, we hypothesized that the impairment of NET degradation might be linked to deficiencies in DNases. We attempted to quantify the functional DNase in patient plasma. We first employed a functional assay to evaluate total functional DNases and then assessed DNase 1 and 1L3 protein levels using ELISA, following current diagnostic strategies for investigating hemostatic disorders. Techniques such as Denaturing Polyacrylamide gel electrophoresis Zymography (DPZ) and Single Radial Enzyme Diffusion (SRED), which necessitates the use of agarose containing dsDNA, have traditionally been used to quantify functional DNase activity, but they are manual methods that require migration and blotting, making them less quantitative, reproducible, and suitable for analyzing a large number of samples. In recent years, new approaches have been developed based on the measurement of DNase activity kinetics, but they require devices with continuous fluorescence recording and precise temperature control throughout the process. Our in-house assay, conducted in liquid conditions allows precise evaluation of the DNase ability to degrade DNA amount from a substantial number of patient samples, while concurrently quantifying NETs.

The quantification of the balance between NET remnants and functional DNase across various severity profiles highlights an inadequate control of NETosis by DNase in hospitalized patients. Our findings corroborate a recent observation by the group of V. Papayannopoulos [23], reporting lower DNase activity and, more specifically, NETase activity in 43 patients with COVID-19 pneumonia. Our study expands on this result by including 145 COVID-19 patients across three distinct severity profiles. Additionally, we show for the first time that the ratios between NET marker levels and functional DNase strongly correlate with markers of disease severity, such as C-Reactive Protein and neutrophil/lymphocyte ratios, further suggesting that inadequate DNase activity may be responsible for elevated NET levels and subsequent clinical deterioration. The question of whether the reduction in DNase amount is specific to COVID-19 remains unclear, as studies on DNase activity in sepsis are scarce. Sohrabipour et al. [24] reported lower DNase1 activity in sepsis, Cox et al. [25] reported diminished serum nuclease activity, and Aramburu et al. [23] recently reported lower DNase and NETase activity in plasma samples from patients with sepsis and COVID-19 pneumonia. This suggests that lower DNase activity may be a common mechanism in severe infections, but further studies are required for a precise assessment, especially given the incomparability of techniques used to measure DNase activity among available studies.

DNase1 and DNase1L3 play pivotal roles in the regulation of DNase activity in plasma, crucial for maintaining the integrity of blood and tissues during sepsis [13]. Notably, in mice lacking both DNase1 and DNase1L3, the induction of septicemia results in intravascular NET clot formation, leading to vessel occlusion and eventual mortality. DNase1 is primarily expressed in non-hematopoietic tissues and exhibits a preference for cleaving protein-free DNA [26, 27]. In the context of sepsis, plasma DNase1 concentrations are known to increase [23]. On the other hand, DNase1L3, often referred to as DNase gamma, is secreted by immune cells and has a specific affinity for DNA–protein complexes, particularly those found in NETs [26, 28]. In our study, we observed higher plasma DNase1 antigen concentrations in non-hospitalized patients compared to healthy controls, consistent with the higher plasma DNase1 concentrations observed in patients with sepsis. Remarkably, we found lower levels of DNase1 antigen in plasma samples from COVID-19 patients who were hospitalized compared to non-severe outpatients, aligning with the lower amount of functional DNase observed in the same patients. Moreover, our analysis of the balance between NET markers and DNase1 or DNase1L3 antigen levels clearly reveals a significant disequilibrium in hospitalized patients compared to non-severe outpatients, favoring an inadequate upregulation of DNase1 and 1L3 secretion. This observation is in line with our findings of a lower functional DNase amount with increased disease severity.

In our genetic analysis, we did not identify any variations in the coding sequence and regulatory regions of *DNASE1L3* that could explain the reduced amounts or functions of DNase1L3 proteins. Our results are consistent with those of Carmona-Rivera et al. [22], who performed whole-genome sequencing in pediatric patients with Multisystem Inflammatory Syndrome in Children and adult patients with COVID-19 and did not find genetic drivers of impaired nuclease activity. In contrast, we discovered polymorphisms in the promoter region of *DNASE1* that were associated with a 75% reduction in DNase1 protein levels. These findings align with data from the GTex portal (https://gtexportal.org/home/index.html), indicating that these polymorphisms are associated with lower *DNASE1* gene expression. However, our study only investigated the association between *DNASE1* and *DNASE1L3* polymorphisms and protein levels. Additional polymorphisms may influence corresponding protein activities, a possibility that cannot be ruled out.

Our research reveals a pronounced dysregulation in the balance between DNase1L3 antigen and the amount of NETs in hospitalized COVID-19 patients. We also observed a reduction in the numbers of immune cells that produce DNase1L3, such as pDCs and DCs. Additionally, we report a lower of *DNASE1L3* in pDCs from the most severe patients. The decrease in circulating pDCs has been previously reported in COVID-19 patients [29–34]. While monocyte subpopulations and conventional DCs migrate from blood to the lungs in severe COVID-19 patients, pDCs were absent from the lungs, indicating a loss of this cell population [30]. Saichi et al. [33] reported several defects in pDC functions (innate sensing, antiviral, and cytotoxic functions). However, the link between the loss of pDCs in such patients and a DNase1L3 deficiency has not been previously described. PDCs are essential sensors of viral infection and produce substantial amounts of interferon in response to SARS-CoV-2, as demonstrated in vitro and ex vivo in non-severe patients [35]. Whether the high levels of self-DNA from NETs induce pDC apoptosis remains to be elucidated. Another potential explanation for the lower DNase1 or DNase1L3 antigen levels could be the presence of autoantibodies against these enzymes, a factor that was not explored in this study. Carmona-Rivera et al. [22] recently reported an association between NET degradation impairment and levels of autoantibodies against DNase1L3, although they did not find such autoantibodies against DNase1.

Our study has some limitations. The first relates to our study design, which combines the analysis of two prospective studies and therefore two different cohorts. This accounts for the variations in the types of samples used (frozen PBMC, DNA) and the inability to quantify DCs and pDCs or search for genetic polymorphisms in *DNASE1* or *DNASE1L3* genes in outpatients. We were also unable to assess the evolution of our markers at the individual level in relation to the natural course of the disease. Additionally, we only had access to blood samples and were unable to investigate the balance between NETs and DNases in tissues, particularly in the lungs, which may differ significantly from blood. Another limitation pertains to the moderate sample size of our study, with potential confounding factors and an imbalance in patients with severe or critical COVID in the groups. Larger studies are needed to confirm the observed association between *DNASE1* polymorphisms and DNase1 protein levels. Furthermore, our technique for quantifying functional DNase measures the amount of protein capable of cleaving double-stranded DNA. This includes DNA originating from lysed cells and NETs. Our technique is therefore not specific for NETs, unlike NETases activity measurements [23, 36]. However, current techniques available for measuring NETase activity lack the capacity to analyze a large number of samples in a reproducible manner. Finally, our technique can only measure the total functional DNases amount, not specifically the activities of DNase1 and DNase1L3. Further studies are necessary to determine the precise contribution of DNases to degrade free DNA and NETs, with specific assessment of DNase1 and DNase1L3 activities in COVID-19 patients with varying disease severity.

In cases where host resistance is compromised or viral aggression persists, an unregulated immune response may lead to organ damage and reduced disease tolerance [37]. Robust NETosis in the early stages of infection may possess beneficial anti-infectious activity, but effective regulatory mechanisms are required. Our study underscores the potential role of a defective upregulation of DNase1 and 1L3 in the accumulation of NETs and clinical deterioration. Notably, the knock-out of either DNase1 or 1L3 in mice is associated with vascular lung occlusion (enriched in NETs) in a neutrophilia sterile model, but the expression of one of the DNases is sufficient to prevent vascular occlusion [13]. Identifying an imbalance between NETosis and DNase activity early in clinical practice may be valuable for proposing appropriate therapies, which could include DNase administration. Administering DNase can currently be achieved through aerosols, and some clinical trials (NCT04359654, NCT04402970, NCT04541979, NCT04445285) have tested inhaled DNase1 administration in severe COVID-19 patients, with some benefits reported [38]. However, inhaled DNase1 may have limited efficacy in severe and critical patients where pulmonary microvessels may already be obstructed by microthrombi. In contrast, administering inhaled DNase1 to patients at an early stage of NET-DNase activity imbalance may be effective in preventing further clinical deterioration or development of long COVID [39]. Our results also encourage the development of systemic DNase administration, either subcutaneously, as recently demonstrated in a mouse model of COVID-19 [40], or intravenously, as proposed in mouse models where increased NETosis is involved.

## Methods

### Study design and setting

This cohort study was conducted from April 2020 to June 2021. The non-severe COVID-19 participant population was recruited from the control arm of the COVERAGE trial, a multi-arm, multistage, randomized controlled adaptive trial (NCT04356495) evaluating the safety, tolerability, and efficacy of various treatment strategies for preventing clinical deterioration in at-risk outpatients with COVID-19. Participants in the control arm received a 10-day course of a cocktail of vitamins and trace elements at physiological doses (Azinc Vitalité^®^) [41, 42]. The severe or critical COVID-19 participant population was recruited from the COLCOV19-BX prospective observational cohort study (NCT04332016).

### Participants

Participants with non-severe COVID-19 were symptomatic adult outpatients consecutively enrolled in the COVERAGE France trial (NCT04356495). Eligibility criteria for this trial included a clinical presentation suggestive of COVID-19 within 7 days, a positive test confirming acute SARS-CoV-2 infection, no need for hospitalization or oxygen therapy, and age 60 or older or between 50 and 59 with additional risk factors for severe disease. These risk factors included conditions such as arterial hypertension under treatment, obesity (BMI ≥ 30 kg/m^2^), diabetes under treatment, ischemic heart disease, heart failure, stroke history, Chronic Obstructive Pulmonary Disease, stage 3 chronic renal failure (30 ≤ Estimated GFR < 60 mL/min/1·73 m^2^), malignancies (solid tumors or blood malignancies), or immunodeficiency. Gender was self-reported by the participants. Participants with severe or critical COVID-19 were consecutively enrolled in the COLCOV19-BX prospective cohort study (NCT04332016) while being hospitalized in a medical unit or an intensive care unit (ICU). Criteria for severity were defined according to WHO classification [43]. Non-severe COVID-19 was defined as the absence of criteria for severe or critical COVID-19. Severe COVID-19 was defined as an oxygen saturation < 90% on room air or signs of severe respiratory distress (accessory muscle use, inability to complete full sentences, respiratory rate > 30 breaths per minute). Critical COVID-19 was defined by the presence of criteria for ARDS, sepsis, septic shock, or other conditions necessitating life-sustaining therapies like mechanical ventilation (invasive or non-invasive) or vasopressor therapy.

### Variables and data measurement

All data were extracted from electronic medical records using various software packages (Metavision (IMDSoft, Wakefield, USA), ICCA (IntelliSpace Critical Care and Anesthesia, Philips Healthcare, Andover, USA) and DxCare (Dedalus, Le Plessis Robinson, France)) and from the COVERAGE study databases. Severity in hospitalized patients was assessed using the SOFA [44] or SAPS II [45] score. Inpatient respiratory parameters included the PaO2/FiO2 ratio, ROX index [18], duration and type of oxygenation (standard or high flow oxygen therapy, invasive or non-invasive ventilation (NIV)), and lung damage on the initial CT scan. Data on thromboembolic events included the presence of deep venous thrombosis, pulmonary embolism, and type of anticoagulation (preventive or curative). Sepsis occurrence was based on the need for antibiotic therapy during the hospital stay and/or sepsis as defined in the 2016 consensus [46]. Collected COVID-19 treatment included antiviral therapy, corticosteroids, convalescent plasma, and tocilizumab.

### Sample preparation

Samples were obtained at the time of inclusion for outpatients in the COVERAGE trial and at the time of admission for inpatients in the COLCOV19-BX cohort study. Plasma samples were collected in citrated tubes for the COVERAGE trial and in citrated and ethylene diamine tetraacetic acid (EDTA) tubes for patients in the COLCOV19-BX cohort. After double centrifugation at 700 g for 10 min, the plasma was aliquoted and frozen at −80 °C within 4 h after blood sampling. Peripheral blood mononuclear cells (PBMC) were separated by centrifugation on a sucrose cushion (Ficoll, LymphoPrep^®^). Red blood cell lysis was performed using Ammonium Chloride Potassium (ACK) buffer (155 mM NH4Cl, 100 μM Na2 EDTA, 10 mM KHCO3). PBMC were washed with phosphate-buffered saline, counted, and frozen in fetal calf serum (FCS) with 10% dimethyl sulfoxide in liquid nitrogen.

### Quantification of NET markers

Based on existing experimental data and previous research [20], several biomarkers of NET were evaluated in citrated plasma: circulating free DNA (cfDNA), extracellular DNA-associated myeloperoxidase (MPO-DNA), citrullinated histone H3 alone or bound to DNA (H3cit and H3cit-DNA). MPO-DNA complexes were quantified using a modified ELISA cell death detection kit approach (ROCHE^®^) with anti-MPO antibody capture and peroxidase-labeled anti-DNA monoclonal antibody for revealing the MPO-DNA complexes (4A4 clone, Bio-Rad AbD Serotec, Kidlington, UK, RRID:AB_617350) and peroxidase-labeled anti-DNA monoclonal antibody (clone MCA-33; 1:20). To limit inter-assay variability, a calibration range was prepared from a stock solution of NETs, and the results are expressed as a percentage of standard NETs (%ST). The stock solution of NETs was prepared from 5 healthy donors (Etablissement Français du Sang) by stimulation with PMA (phorbol 12-myristate 13-acetate). H3cit was quantified according to a slight modification of the enzyme-linked immunosorbent assay previously described by Thalin and colleagues [47], using an anti-Histone antibody capture (MAB3422, clone H11-4; Sigma-Aldrich, RRID:AB_2114845) and an anti-H3cit antibody (citrulline R2,R8,17, ab5103; Abcam Cambridge, UK, RRID:AB_304752). DNA-H3cit was quantified using an enzyme-linked immunosorbent assay previously described by Thalin et al. [48] using the capture anti-H3Cit antibody (ab232939; Abcam Cambridge, UK). Results were expressed as a fold change between the patient's sample absorbance and the mean absorbance of the healthy control group.

### Functional DNases assay

To assess the functionality of circulating DNases, a fluorometrically based in-house functional DNase assay adapted in a microplate was performed using citrated plasma. This assay is based on DNase-induced double stranded DNA (dsDNA) hydrolysis and measurement of residual dsDNA with a fluorescent intercalating agent (PicoGreen^™^). Two adjacent wells were used, one containing 50 ng of dsDNA standard as a substrate (DNA( +)) and the other containing buffer (DNA(-)). A reference well is used and contain 50 ng of dsDNA. dsDNA is lambda DNA provided in the Quant-it TM PicoGreen TM dsDNA kit (ThermoFisher Scientific). Sample introduction and incubation were carried out for two hours at 37 °C. The reaction was stopped using PBS-EGTA 25 mM, and PicoGreen solution was added to evaluate residual DNA. DNases functional activity was expressed as the percentage of DNA degraded using a specific formula. Results were expressed as a fold change between the patient's sample absorbance and the mean absorbance of the healthy control group.

### DNase 1L3 and DNase 1 antigen quantification

Quantification of DNase1 and DNase1L3 antigens was performed using commercial ELISA kits (LSDbio kit^®^ and AVIVA kit^®^). EDTA plasma was used for hospitalized patients, and citrated plasma was used for outpatients. To enable comparison between the two patient populations with different blood samples, DNase1 and DNase1L3 antigens from healthy donors were measured in both conditions. Results were expressed as a fold change between the patient's sample absorbance and the mean absorbance of the healthy control group.

### *DNASE1 and DNASE1L3* polymorphisms

Sanger sequencing of *DNASE1* and *1L3* genes was performed on peripheral blood from 52 inpatients using a commercial DNA extraction kit (QIAmp DNA preparation kit; Qiagen SA). Sequencing was carried out using primers designed to span the 5’UTR region and all the coding regions, including the intron–exon boundaries (sequences and amplification conditions are described in Additional file 1: Tables S1 and S2). Prior to sequencing, excess primers and nucleotides were removed with enzymatic purification using ExoSAP-ITTM-PCR product cleanup reagent provided by Applied Biosystems. Direct sequencing of purified PCR products was assessed by the dideoxynucleotide chain termination method using the ABI Prism^™^ BigDye^™^ Terminator Cycle Sequencing Ready Reaction Kit on an ABI Prism 3500xL Genetic analyzer (Applied Biosystems, Foster City, CA) and the sequences were analyzed by the SeqScape Sofware (Applied Biosystems, Foster City, CA). *DNASE1* and *DNASE1L3* variants were annotated according to the reference genome GRCh38 GenBank NM_005223.4 and NM_004944.4, respectively. Sequence variations were numbered according to the Human Genome Organization recommendation (http://.hgvs.org), and Alamut was used to assess putative consequences of mutations.

### DNASE1 and 1L3 single-cell RNA-seq gene expression analysis

Publicly available data from the study by Stephenson and colleagues [19] were used to analyze single-cell RNA-seq gene expression of *DNASE1* and *1L3*. The data were processed by the authors and were downloaded from https://www.covid19cellatlas.org/. The Seurat R package was employed to visualize *DNASE1* and *1L3* normalized gene expression in different cell subsets and donors.

### Flow cytometry of dendritic cells

Frozen PBMC were thawed in RPMI-50% FCS, counted in Trypan blue, and 1 × 10^6^ cells were stained with a viability dye (Viobility 405/520, Fixable Dye, Miltenyi-Biotech), Lineage antibodies (anti-CD3-BV510 (BD Biosciences), CD19-VioGreen (Miltenyi-Biotech) and CD56-BV510 (BioLegend)), and anti-CD14-BV650 (Biolegend), HLA-DR VioBlue (Miltenyi-Biotech), CD141-BV785 (BioLegend), CD16-PE (Beckman-Coulter), CD1c-BV605 (BD Biosciences), FcERI-PerCP-Vio700 (Miltenyi-Biotech), CD123-APC-Vio770 (Miltenyi-Biotech), CD11c-PE-Vio615 (Miltenyi-Biotech) antibodies (all antibodies are described in Additional file 1: Table S3). Samples were processed on the BD LSRFortessa cytometer (BD Biosciences). pDC and DC cells were identified as described by Mair et al. [49]. Data were analyzed using FlowJoTM version v10·8 software (BD Life Sciences).

### Statistical analysis

The study's sample size was determined based on preliminary data. We estimated that 23 patients should be enrolled in each group to allow a 90% probability that the study would detect a treatment difference at a two-sided significance level of 0.05. In the outpatient group, we considered that 25% of patients would require hospitalisation for oxygen therapy, as we began this study at the start of the pandemic. In order to determine the prognostic value of the markers studied, we therefore estimated that 92 patients should be included in the outpatient group. Categorical variables are expressed as numbers and percentages. Continuous variables are expressed as mean and standard deviation (SD) or median and interquartile range (IQR), depending on the normality of their distribution, assessed using the Kolmogorov–Smirnov test.

Because of the semi-continuous nature of NETs and DNases biomarkers, we employed the Compound Poisson-Gamma model [50], as implemented in the cplm R package, to assess their association with COVID-19 severity. Correlations with CRP, neutrophil/lymphocytes ratio, P/F ratio and ROXX index were assessed using the Spearman correlation coefficient. All statistical analyses were adjusted for age, sex and BMI.

Statistical significance was set at p < 0.05, and analyses were performed using the Rstudio environment (Version 1.3.1093, © 2009–2020 RStudio, PBC, Boston). Figures were realized using GraphPad Prism version 6.00 software (GraphPad Software, La Jolla, CA, USA). Receiver operating characteristic (ROC) curves were used to identify parameters predicting hospitalization of COVID-19 outpatients, and analyses were performed using SAS (SAS Institute, Cary, NC, USA) and GraphPad Prism version 6.00 software (GraphPad Software, La Jolla, CA, USA). Association of identified *DNASE1* and *1L3* polymorphisms with the plasma activity of their associated proteins was tested using regression models adjusting for age and gender. For *DNAse1L3*, associations were conducted using linear regression analysis on log-transformed values. For *DNAse1* that exhibited a semi-continuous distribution a Compound Poisson-Gamma model [50] was employed. Genetic association analyses were restricted to polymorphisms with rare alleles present at least in 3 patients, corresponding to an allele frequency of ~ 3%. For these analyses, the Rstudio environment (Version 1.3.1093, © 2009–2020 RStudio, PBC, Boston) was used.

### Study approval

Both studies were promoted by the Bordeaux University Hospital and received the necessary approvals from ethics committees. The COVERAGE trial protocol was approved by a French Ethics Committee (CPPIDF1-2020-ND45) and the French Medicine Agency (MEDAECNAT-2020-03-00065). The COLCOV trial was approved by a French Ethics Committee (CPPI DF3: ref 3791-RM). Written informed consent was obtained from all participants in the COVERAGE and COLCOV studies, and the trials were conducted in compliance with ethical approval.

### Supplementary Information

**Additional file 1: Figure S1.** CfDNA according to COVID 19 clinical severity (a); ratios between cfDNA and functional DNase (b), DNase1 protein (c) and DNase1L3 protein (d). Results expressed in fold change and fold change ratio between non-serve (n = 32), severe (n = 15) and critical COVID-19 patients (n = 37) and healthy donors (n-7). Comparisons between groups were performed using a Compound Poisson-Gamma model adjusted for age, sex and BMI. **Figure S2.** Spearman correlation adjusted for age, sex and BMI between three NET biomarkers, MPO-DNA (a, d), H3cit (b, e), and H3cit-DNA (c, f) and two markers of disease severity, i.e. the Pao2/FiO2 ratio (a–c) and the ROX index (d–f) (n = 52). **Figure S3.** Spearman correlation adjusted for age, sex and BMI between the ratios of three biomarkers, MPO-DNA (a, d), H3cit (b, e), and H3cit-DNA complexes (c, f) over functional DNase and two markers of disease severity, i.e. the PaO2/FiO2 ratio (a–c) and the ROX index (d–f) (n = 52). **Figure S4.** Gating strategy for dendritic cells (cDC1 and cDC2) and plasmacytoid dendritic xells (pDCs) analysis. DC were selected from living Lin-cells after basophil exclusion, as CD14- and CD16-. Among DCs, pDC were defined as CD123 + CD11c- cells and cDC as CD123-CD11c + cells. cDC1 and cDC2 subpopulations were discriminated by their expression of CD141 and CD1c. **Table S1.** Primers used for DNASE1 genotyping. **Table S2.** Primers used for DNASE1L3 genotyping. **Table S3.** Antibodies used for Flow Cytometry experiments. **Table S4.** List of DNASE1L3 polymorphisms identified in hospitalized patients. **Table S5.** List of DNASE1 polymorphisms identified in hospitalized patients.

## Data Availability

Data are available in the Additional file 1 and from the corresponding author upon request. RNA-Seq data are available on the public database https://www.covid19cellatlas.org/ [19].

## References

[1] Petersen E (2020). Comparing SARS-CoV-2 with SARS-CoV and influenza pandemics. Lancet Infect Dis.

[2] Wiersinga WJ (2020). Pathophysiology, transmission, diagnosis, and treatment of coronavirus disease 2019 (COVID-19): a review. JAMA.

[3] Zhu N (2020). A novel coronavirus from patients with pneumonia in China, 2019. N Engl J Med.

[4] Acute Respiratory Distress Syndrome (2012). The Berlin definition. JAMA.

[5] Wu C (2020). Risk factors associated with acute respiratory distress syndrome and death in patients with coronavirus disease 2019 pneumonia in Wuhan, China. JAMA Intern Med.

[6] Karagiannidis C (2020). Case characteristics, resource use, and outcomes of 10 021 patients with COVID-19 admitted to 920 German hospitals: an observational study. Lancet Respir Med.

[7] Matuozzo D (2023). Rare predicted loss-of-function variants of type I IFN immunity genes are associated with life-threatening COVID-19. Genome Med.

[8] Bastard P (2020). Autoantibodies against type I IFNs in patients with life-threatening COVID-19. Science.

[9] Lamers MM, Haagmans BL (2022). SARS-CoV-2 pathogenesis. Nat Rev Microbiol.

[10] Bonaventura A (2021). Endothelial dysfunction and immunothrombosis as key pathogenic mechanisms in COVID-19. Nat Rev Immunol.

[11] Engelmann B, Massberg S (2013). Thrombosis as an intravascular effector of innate immunity. Nat Rev Immunol.

[12] Brinkmann V (2004). Neutrophil extracellular traps kill bacteria. Science.

[13] Jiménez-Alcázar M (2017). Host DNases prevent vascular occlusion by neutrophil extracellular traps. Science.

[14] Charlotte T (2019). Neutrophil extracellular traps. Arterioscler Thromb Vasc Biol.

[15] McFadyen JD, Stevens H, Peter K (2020). The emerging threat of (micro) thrombosis in COVID-19 and its therapeutic implications. Circ Res.

[16] Zuo Y (2021). Autoantibodies stabilize neutrophil extracellular traps in COVID-19. JCI Insight.

[17] Wang Q (2021). Clinical value of laboratory indicators for predicting disease progression and death in patients with COVID-19: a retrospective cohort study. BMJ Open.

[18] Roca O (2019). An index combining respiratory rate and oxygenation to predict outcome of nasal high-flow therapy. Am J Respir Crit Care Med.

[19] Cambridge Institute of Therapeutic Immunology and Infectious Disease-National Institute of Health Research (CITIID-NIHR) COVID-19 BioResource Collaboration (2021). Single-cell multi-omics analysis of the immune response in COVID-19. Nat Med.

[20] Prével R (2022). Plasma markers of neutrophil extracellular trap are linked to survival but not to pulmonary embolism in COVID-19-related ARDS patients. Front Immunol.

[21] Masuda S (2016). NETosis markers: quest for specific, objective, and quantitative markers. Clin Chim Acta.

[22] Carmona-Rivera C (2022). Multicenter analysis of neutrophil extracellular trap dysregulation in adult and pediatric COVID-19. JCI Insight.

[23] Aramburu IV (2022). Functional proteomic profiling links deficient DNA clearance with increased mortality in individuals with severe COVID-19 pneumonia. Immunity.

[24] Sohrabipour S (2021). Mechanistic studies of DNase I activity: impact of heparin variants and PAD4. Shock.

[25] Cox LE (2020). Neutrophil extracellular trap formation and nuclease activity in septic patients. BMC Anesthesiol.

[26] Napirei M (2009). Murine serum nucleases – contrasting effects of plasmin and heparin on the activities of DNase1 and DNase1-like 3 (DNase1l3). FEBS J.

[27] Napirei M (2004). Expression pattern of the deoxyribonuclease 1 gene: lessons from the Dnase1 knockout mouse. Biochemical Journal.

[28] Sisirak V (2016). Digestion of chromatin in apoptotic cell microparticles prevents autoimmunity. Cell.

[29] Zhou R (2020). Acute SARS-CoV-2 infection impairs dendritic cell and T cell responses. Immunity.

[30] Sánchez-Cerrillo I (2020). COVID-19 severity associates with pulmonary redistribution of CD1c+ DCs and inflammatory transitional and nonclassical monocytes. J Clin Investig.

[31] Peruzzi B (2020). Quantitative and qualitative alterations of circulating myeloid cells and plasmacytoid DC in SARS-CoV-2 infection. Immunology.

[32] Laing AG (2020). A dynamic COVID-19 immune signature includes associations with poor prognosis. Nat Med.

[33] Saichi M (2021). Single-cell RNA sequencing of blood antigen-presenting cells in severe COVID-19 reveals multi-process defects in antiviral immunity. Nat Cell Biol.

[34] Kulkarni-Munje A (2021). Disease-duration based comparison of subsets of immune cells in SARS CoV-2 infected patients presenting with mild or severe symptoms identifies prognostic markers for severity. Immunity Inflam Dis.

[35] Severa M (2021). Differential plasmacytoid dendritic cell phenotype and type I interferon response in asymptomatic and severe COVID-19 infection. PLoS Pathog.

[36] Englert H (2023). Targeting NETs using dual-active DNase1 variants. Front Immunol.

[37] Ayres JS (2020). Surviving COVID-19: a disease tolerance perspective. Sci Adv.

[38] Holliday ZM (2021). Non-randomized trial of dornase alfa for acute respiratory distress syndrome secondary to covid-19. Front Immunol.

[39] Krinsky N (2023). NETosis induction reflects COVID-19 severity and long COVID: insights from a 2-center patient cohort study in Israel. J Thromb Haemost.

[40] Veras FP (2023). Targeting neutrophils extracellular traps (NETs) reduces multiple organ injury in a COVID-19 mouse model. Respir Res.

[41] Duvignaud A (2022). Inhaled ciclesonide for outpatient treatment of COVID-19 in adults at risk of adverse outcomes: a randomised controlled trial (COVERAGE). Clin Microbiol Infect.

[42] Duvignaud A (2020). Home Treatment of Older People with Symptomatic SARS-CoV-2 Infection (COVID-19): a structured Summary of a Study Protocol for a Multi-Arm Multi-Stage (MAMS) Randomized Trial to Evaluate the Efficacy and Tolerability of Several Experimental Treatments to Reduce the Risk of Hospitalisation or Death in outpatients aged 65 years or older (COVERAGE trial). Trials.

[43] WHO (2022). Clinical management of COVID-19: living guideline.

[44] Vincent J-L (1996). The SOFA (Sepsis-related Organ Failure Assessment) score to describe organ dysfunction/failure: on behalf of the working group on sepsis-related problems of the european society of intensive care medicine (see contributors to the project in the appendix). Intensive Care Med.

[45] Gall J-RL (1984). A simplified acute physiology score for ICU patients. Crit Care Med.

[46] Singer M (2016). The third international consensus definitions for sepsis and septic shock (Sepsis-3). JAMA.

[47] Thålin C (2017). Validation of an enzyme-linked immunosorbent assay for the quantification of citrullinated histone H3 as a marker for neutrophil extracellular traps in human plasma. Immunol Res.

[48] Thålin C (2020). Quantification of citrullinated histones: development of an improved assay to reliably quantify nucleosomal H3Cit in human plasma. J Thromb Haemost.

[49] Mair F, Liechti T (2021). Comprehensive phenotyping of human dendritic cells and monocytes. Cytometry Pt A.

[50] Zhang Y (2013). Likelihood-based and Bayesian methods for Tweedie compound Poisson linear mixed models. Stat Comput.

