# Supplementary material for: Impaired balance between neutrophil extracellular trap formation and degradation by DNases in COVID-19 disease
Source: J Transl Med. 2024 Mar 7;22:246. doi: 10.1186/s12967-024-05044-7 (PMC10919029; doi:10.1186/s12967-024-05044-7)
Supplement: Supplementary file 1 — Additional file 1: Figure S1. CfDNA according to COVID 19 clinical severity (a); ratios between cfDNA and functional DNase (b), DNase1 protein (c) and DNase1L3 protein (d). Results expressed in fold change and fold change ratio between non-serve (n = 32), severe (n = 15) and critical COVID-19 patients (n = 37) and healthy donors (n-7). Comparisons between groups were performed using a Compound Poisson-Gamma model adjusted for age, sex and BMI. Figure S2. Spearman correlation adjusted for age, sex and BMI between three NET biomarkers, MPO-DNA (a, d), H3cit (b, e), and H3cit-DNA (c, f) and two markers of disease severity, i.e. the Pao2/FiO2 ratio (a–c) and the ROX index (d–f) (n = 52). Figure S3. Spearman correlation adjusted for age, sex and BMI between the ratios of three biomarkers, MPO-DNA (a, d), H3cit (b, e), and H3cit-DNA complexes (c, f) over functional DNase and two markers of disease severity, i.e. the PaO2/FiO2 ratio (a–c) and the ROX index (d–f) (n = 52). Figure S4. Gating strategy for dendritic cells (cDC1 and cDC2) and plasmacytoid dendritic xells (pDCs) analysis. DC were selected from living Lin-cells after basophil exclusion, as CD14- and CD16-. Among DCs, pDC were defined as CD123 + CD11c- cells and cDC as CD123-CD11c + cells. cDC1 and cDC2 subpopulations were discriminated by their expression of CD141 and CD1c. Table S1. Primers used for DNASE1 genotyping. Table S2. Primers used for DNASE1L3 genotyping. Table S3. Antibodies used for Flow Cytometry experiments. Table S4. List of DNASE1L3 polymorphisms identified in hospitalized patients. Table S5. List of DNASE1 polymorphisms identified in hospitalized patients. [file 12967_2024_5044_MOESM1_ESM.pdf]

Supplementary Figure 1 –cfDNA according to COVID 19 clinical severity (a); ratios between cfDNA and functional DNase (b), DNase1 protein (c) and DNase1L3 protein (d). Results expressed in fold change and fold change ratio between non-severe (n = 32), severe (n = 15) and critical COVID-19 patients (n = 37) and healthy donors (n = 7). Comparisons between groups were performed using a Compound Poisson-Gamma model adjusted for age, sex and BMI.

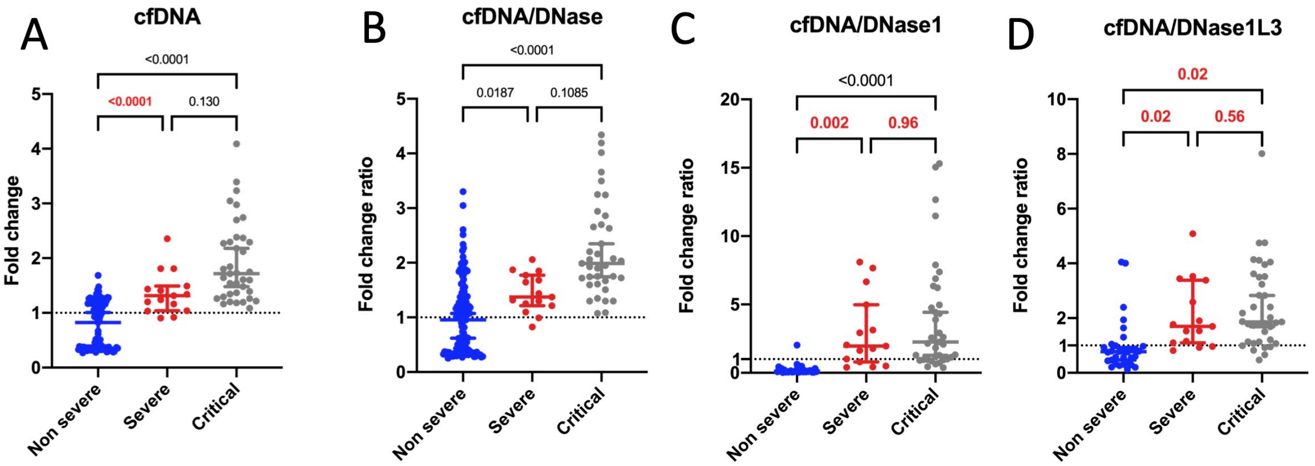

Supplementary Figure 2 – Spearman correlation **adjusted for age, sex and BMI** between three NET biomarkers, MPO-DNA (a, d), H3cit (b, e), and H3cit-DNA (c, f) and two markers of disease severity, *i.e.* the PaO2/FiO2 ratio (a-c) and the ROX index (d-f) (n =52).

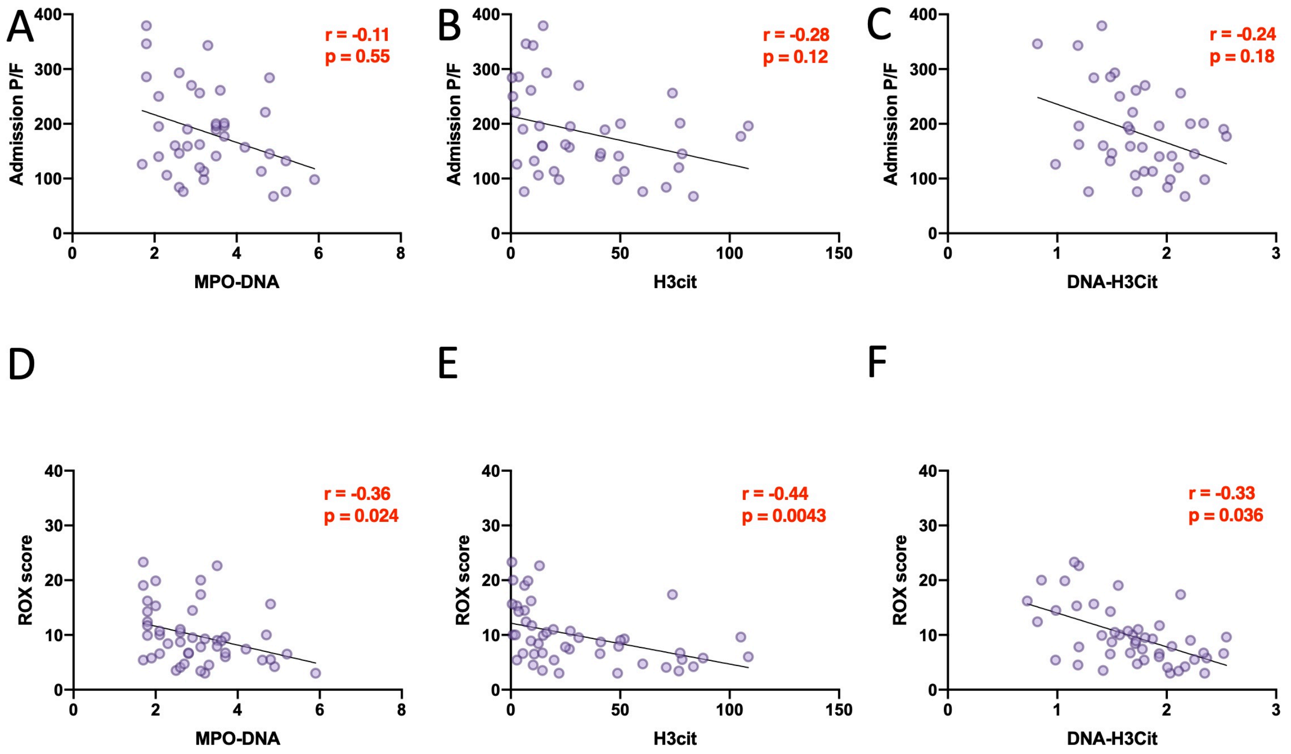

Supplementary Figure 3 – Spearman correlation **adjusted for age, sex and BMI** between the ratios of three NET biomarkers, MPO-DNA (a, d), H3cit (b, e), and H3cit-DNA complexes (c, f) over functional DNase and two markers of disease severity, *i.e.* the PaO2/FiO2 ratio (a-c) and the ROX index (d-f) (n =52).

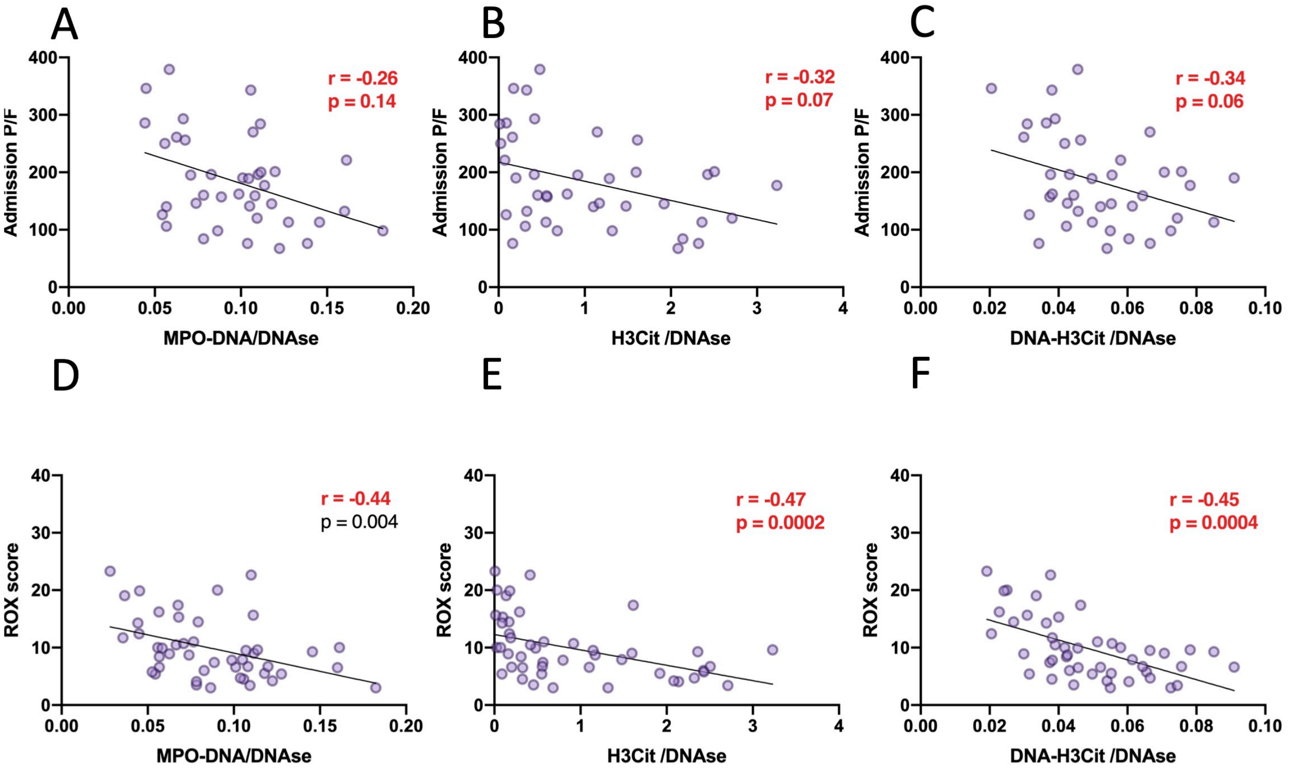

Supplementary Figure 4 – Gating strategy for dendritic cells (cDC1 and cDC2) and plasmacytoid dendritic cells (pDCs) analysis. DC were selected from living Lin- cells after basophil exclusion, as CD14- and CD16-. Among DCs, pDC were defined as CD123+CD11c- cells and cDC as CD123-CD11c+ cells. cDC1 and cDC2 subpopulations were discriminated by their expression of CD141 and CD1c.

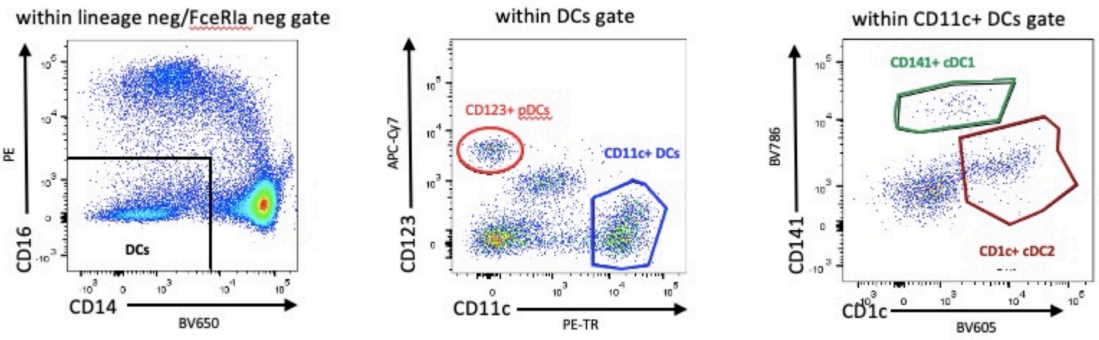

***Supplemental Table 1** – Primers used for DNASE1 genotyping*

**DNASE1**<br>**Accession** NG_009285 **Transcript_ID** NM_005223.3						
**ID**		**Sequence (5'->3')**	**Length**	**Tm**	**GC%**	**Product length**
DNASE1_5UTR	F	TCCTGACTTTCGACCTTGGC	20	59.97	55.00	650
	R	ACCCCGCCACTCATTTCTTG	20	60.61	55.00	
DNASE1_EX1a	F	CTTCAGACGTCCAGTCTCCG	20	59.83	60.00	872
	R	CCAGGCCTCAGGTCCAGTAA	20	60.91	60.00	
DNASE1_EX1b	F	CCAAGAAATTACTGGACCTGAGGC	24	61.40	50.00	522
	R	AAAATGTCTGATAACAAGGCCAAAG	25	58.54	36.00	
DNASE1_EX1c	F	ACGGGATAGGAACCTTTGGC	20	59.74	55.00	614
	R	TCCTACAACGTCCAGAAAGCC	21	60.00	52.38	
DNASE1_EX2	F	GTTTGGCTTTCTGGACGTTGT	21	59.60	47.62	656
	R	ATGTCATAGCGGCTCAGGATCT	22	61.09	50.00	
DNASE1_EX3-4	F	CTTTGTGGCGCTGTAGGGT	19	60.30	57.89	482
	R	TAGGACAAACTACTTCCCTGCTC	23	59.49	47.83	
DNASE1_EX5-6	F	CTCCTGGGAAGCAGGAGTG	19	60.9	63.2	674
	R	CTGGGAGGGTCTCACATAGC	20	59.7	60	
DNASE1_EX7-8	F	ATGTCCAAGAGAAATGGGGCT	21	59.36	47.62	799
	R	GTCTGGGCTGAGCCTACCTG	20	61.97	65.00	
DNASE1_EX9	F	GTATGTGTCCTCCCTTGCACA	21	60.00	52.38	364
	R	CATGGTGGGCCGCTTGAAT	19	61.05	57.89	

Supplemental Table 2 *– Primers used for DNASE1L3 genotyping*

**DNASE1L3**<br>**Accession** NG_032070 **Transcript_ID** NM_004944.4						
**ID**		**Sequence (5'->3')**	**Length**	**Tm**	**GC%**	**Product length**
DNASE1L3_EX1	F	TCCTCAGAGAACTGAAAGTTGC	22	58.27	45.45	415
	R	CAAGCCAGCTTCTACATTCCC	21	58.98	52.38	
DNASE1L3_EX2	F	CACTCAGTGCAATACTTAAAAACAG	25	57.16	36.00	311
	R	CAACACTGGCTGTGTGAACTG	21	59.94	52.38	
DNASE1L3_EX3	F	GCGTATGGAGGCAGATTGGA	20	59.89	55.00	325
	R	AGGCCCCTAGTTTTGACAGC	20	59.96	55.00	
DNASE1L3_EX4	F	AGAAAGCCATGGGAACCTACA	21	59,01	47,62	353
	R	CACTGAACTGCATCGTCTACATTG	24	60,2	45,83	
DNASE1L3_EX5	F	CTGGTTTCCCTCCAGCAGTGT	21	62.27	57.14	337
	R	GCTGAAGAAAGGCCTTAAAGAATGC	25	61.13	44.00	
DNASE1L3_EX6	F	TCAGCCCATAACAGACCCTG	20	59.09	55.00	695
	R	ACATTTAATTGACTCAGCTCTGC	23	57.44	39.13	
DNASE1L3_EX7	F	GAACCATGGCAGGGACTGAT	20	59.74	55.00	633
	R	AGGGTCAAGGTCTAGTGCCC	20	60.91	60.00	
DNASE1L3_EX8	F	CCTCCCTGGTCCTAAATTTGC	21	58.62	52.38	768
	R	GATGACACTGATGCTTCCTGG	21	58.71	52.38	

Supplemental Table 3 *– Antibodies used for Flow Cytometry experiments*

**Fluorochrome**	**Marker**	**Company**	**Clone**	**Dilution**	**Purpose**
BV510	CD3	BD Bioscience	UCHT1/563109	1:100	T cells
VioGreen	CD8	Miltenyi	REA734	1:100	T cells
VioGreen	CD19	Miltenyi	REA675	1:100	B cells
BV510	CD56	Biolegend	HCD56	1:100	NK cells
405/520	Viability	Miltenyi	-	1:1000	Viability
PerCP-700	FCeRIa	Miltenyi	REA758	1:100	Basophils
BV650	CD14	Biolegend	M5E2	3:100	Monocytes
PE	CD16	Beckman Coulter	A07766	1:25	Monocytes
APC-Vio 770	CD123	Miltenyi	REA918	1:100	pDCs
PE-Vio 615	CD11c	Miltenyi	REA618	1:100	DCs
BV785	CD141	Biolegend	M80	1:100	cDC1s
BV605	CD1c	BD	F10/21A3	1:100	cDC2s
VioBlue	HLA-DR	Miltenyi	REA805	1:20	

***Supplemental Table 4** – List of DNASE1L3 polymorphisms identified in hospitalized patients*

rsID	Genomic position (GRCh38)	Alleles[1]	Amino Acid position	Localization
rs2292677	58210912	c.-6G>A		5'UTR
rs34252389	58210856	c.51C>T		Exon 1
rs3732631	58208324	c.142-18G>T		Intron 1
rs74350392	58205547	c.244G>C	p.Gly82Arg	Exon 3
rs2070117	58205539	c.252G>A	p.Thr84=	Exon 3
rs17058970	58205515	c.276G>C	p.Arg92=	Exon 3
rs3772985	58200992	c.546+5G>T		Intron 5
rs35677470	58197909	c.616C>T	p.Arg206Cys	Exon 6
rs113844064	58197694	c.704+127G>T		Intron 6
rs1210058558[2]	58193474	c.705-35G>A		Intron 6
rs3732630	58193301	c.801+42A>G	p.Lys281=	Intron 7
rs144058112	58193270	c.801+73T>C		Intron 7
rs3732629	58193139	c.801+204G>A		Intron 7
rs34882513	58192419	c.*268delC		3' UTR

[1] : Nomenclature according to NM_004944.4 reference.
[2] : This variant is present in less than 3 individuals in our cohort

Supplemental Table 5 *– List of DNASE1 polymorphisms identified in hospitalized patients*

rsID	Genomic position (GRCh38)	Alleles[1]	Amino Acid position	Localization
NA[2]	3653234	c.-1812C>T	NA	Upstream transcript
rs45564535	3653249	c.-1797A>C	NA	Upstream transcript
rs45606645	3653474	c.-1572T>C	NA	Upstream transcript
rs45626736	3653508	c.-1538G>A	NA	Upstream transcript
rs111733271[2]	3653575	c.-1471G>A	NA	Upstream transcript
NA[2]	3653620	c.-1426A>T	NA	Upstream transcript
NA[2]	3653653	c.-1393A>T	NA	Upstream transcript
rs960348066[2]	3653835	c.-1211A>C	NA	Upstream transcript
rs865833716	3653840	c.-1206A>C	NA	Upstream transcript
rs867920095	3653841	c.-1205A>G	NA	Upstream transcript
rs77563984	3653879	c.-1167C>T	NA	Upstream transcript
rs79356805	3653880	c.-1166C>G	NA	Upstream transcript
rs45522434[2]	3654385	c.-661C>A	NA	Upstream transcript
rs17136471	3654596	c.-450G>A	NA	Upstream transcript
rs45479597[2]	3654737	c.-309G>A	NA	5UTR
NA	3654725_ 3654729	c.-317_-313del	NA	5UTR
rs769858733[2]	3655050	c.-2+6C>T		Intron 1
rs117176134	3655097	c.-2+54G>C		Intron 1
NA2	3655333_3655336	c.-1-40_-1-38del		Intron 1
rs8176922	3655358	c.-1-15C>T		Intron 1
rs34907394[2]	3655478	c.105G>C	p.Glu35Asp	Exon 2
rs146417970	3655625	c.147+105T>C		Intron 2
rs181314680	3655818	c.148-31C>G		Intron 2
rs143058517[2]	3656648	c.331G>A	p.Val111Met	Exon 5
rs34923865[2]	3656667	c.350A>C	p.Tyr117Ser	Exon 5
NA[2]	3656722	c.405A>G	p.(Pro135=)	Exon 5
rs8176938[2]	3657090	c.528C>G	p.Val176=	Exon 6
NA[2]	3657115	c.556A>T	p.(Met186Leu)	Exon 7
rs1452101461[2]	3657326	c.689A>G	p.His230Arg	Exon 7
rs1799892	3657408	c.704+67G>C		Intron 7
rs1053874	3657746	c.731G>A	p.(Arg244Gln)	Exon 8
NA[2]	3657788	c.773A>T	p.(Gln258Leu)	Exon 8

[1] : Nomenclature according to NM_005223.4 reference
[2] : This variant is present in less than 3 individuals in our cohort
